# An enemy in shadows—Mycoplasma hominis septic arthritis and iliopsoas abscess: Case report and review of the literature

**DOI:** 10.1016/j.idcr.2021.e01260

**Published:** 2021-08-24

**Authors:** Gawahir A. Ali, Wael Goravey, Abdulrahman Hamad, Emad B. Ibrahim, Mohamed R. Hasan, Muna Al Maslamani, Hussam Al Soub

**Affiliations:** a Department of Infectious Diseases, Communicable Diseases Centre, Hamad Medical Corporation, Doha, Qatar; b Department of Internal Medicine, Hamad Medical Corporation, Doha, Qatar; c Department of Laboratory Medicine and Pathology, HMC, Doha and Qatar University, Biomedical Research Centre, Doha, Qatar; d Department of Pathology, Sidra Medicine, Doha, Qatar

**Keywords:** Mycoplasma hominis, septic arthritis, rituximab, PCR, doxycycline

## Abstract

Mycoplasma hominis (M. hominis) is fastidious and difficult to grow bacteria with the ability to colonize the genitourinary and respiratory tracts. Infrequently can cause a variety of genitourinary tract infections, pregnancy complications, and neonatal diseases. M. hominis rarely reported to cause extragenital infections and seldomly native joint septic arthritis particularly in immunocompromised hosts, raising diagnostic challenges and is often associated with delayed diagnosis and high morbidity and mortality. We report the case of a 30-year-old patient who developed M. hominis native left hip septic arthritis with iliopsoas abscess after receiving rituximab for newly diagnosed thrombotic thrombocytopenic purpura (TTP). The diagnosis of M. hominis hip septic arthritis with iliopsoas involvement was confirmed following repeated joint and abscess aspiration and identification of the organism with the aid of culture and specific Polymerase chain reaction (PCR). The patient was subsequently treated with a prolonged course of antibiotics targeting the organism with a favorable outcome. The clinical presentations, assessment, and management of this rare entity of M. hominis related extragenital infections are outlined. In addition, the literature on similar cases was reviewed to raise awareness and avoid devastating consequences.

## Highlights

• The pathogenic potential of Mycoplasma hominis.
• The diagnostic challenges of M. hominis extragenital infections.
• M. hominis septic arthritis, particularly in immunocompromised hosts needs to be considered.
• Molecular methods have revolutionized the detection of M. hominis and can prevent devastating consequences.
• Prolonged antibiotic therapy targeting M. hominis in addition to appropriate surgical interventions is the mainstay of management.

## Introduction

M. hominis belongs to the Mycoplasmataceae family within the Mollicutes class, the smallest and simplest self-replicating free-living bacteria. It constitutes one of the genital mycoplasma species capable of causing human infections which include Ureaplasma urealyticum, Ureaplasma parvum, and Mycoplasma genitalium [1]. M. hominis is part of the flora of the genitourinary tract of healthy individuals with colonization rates ranging between 21% and 53%. However, it is potentially pathogenic and can cause genitourinary, maternal, and neonatal infections [2].

Rarely, M. hominis can cause a wide variety of extragenital infections including native joint septic arthritis, particularly in immunocompromised hosts. Many immunocompromising conditions have been implicated as risk factors for the development of septic arthritis, but most importantly low IgG level seems to be a major determinant [3].

Characteristically slow-progressing infections with repeatedly negative cultures and subsequent joint destruction are frequently observed if not recognized and treated promptly.

The clinical presentation is usually undistinguished M. hominis septic arthritis from typical infective arthritis raising the need for highly sensitive and specific identification methods [4].

The slow and fastidious growth habits of M. hominis limit the timely diagnosis of acute infection by conventional culture methods and lack of cell wall hinder detection by gram staining from the affected joints. Many molecular methods have been developed to avoid delaying the diagnosis, including Real-time PCR and specific Mycoplasma homins 16Sr RNA [2].

Usually, a combination of prolonged antibiotic therapy targeting the organism in addition to appropriate surgical interventions such as arthroscopy, joint washout, or abscess drainage is the mainstay of management [4].

In the present report, we describe a case of Mycoplasma hominis native hip septic arthritis with iliopsoas abscess, which was successfully treated with a combination of antibiotics and surgical intervention and we reviewed the literature for similar cases.

## Case description

**Fig. 1:**
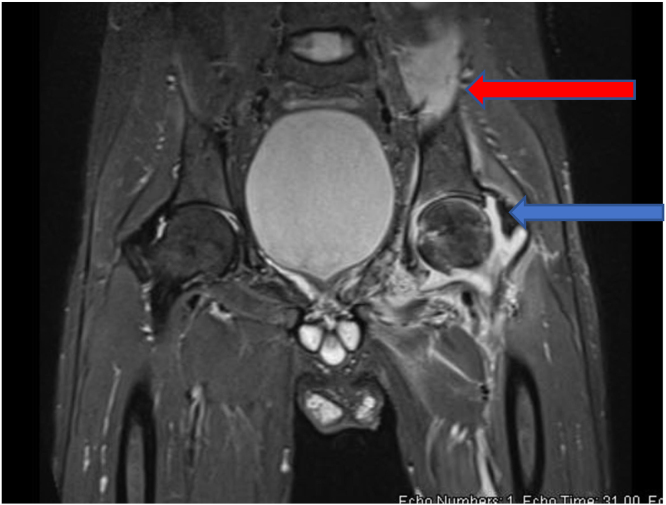
MRI of the left hip demonstrating synovial thickening (Blue) and inflammatory changes involving the surrounding muscles including the iliopsoas (Red). For interpretation of the references to color in this figure legend, the reader is referred to the web version of this article.

**Fig. 2:**
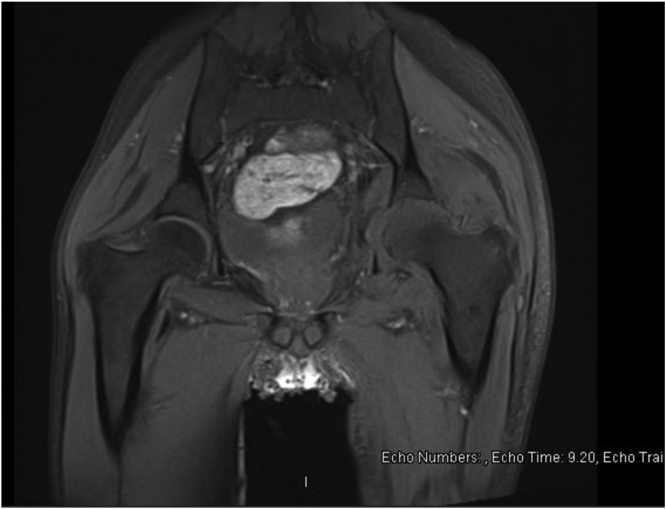
Follow up MRI of the left hip showing appreciable interval decrease in the synovial thickening and inflammatory changes involving the surrounding muscles.

A 30-year-old male presented acutely to our medical service with a petechial rash over his body of 2-day duration. He had no chronic medical conditions and works as a waiter. Further evaluation revealed a diagnosis of refractory TTP and received plasma exchange, steroid, Rituximab, and Caplacizumab with excellent recovery. Through this admission, he developed a seizure with suggestive MRI of neurocysticercosis and received Albendazole with steroids. Furthermore, he developed MSSA bacteremia likely lines related and started on Cefazolin with the fever subsided and no evidence of endocarditis on the Echocardiogram. Toward the end of the two weeks course of Cefazolin and 4 weeks of hospitalization, the fever recurred with left hip pain. On examination, the left hip was held in flexion, externally rotated, and exquisitely painful with minimal movement in all directions. Laboratory findings revealed a white cell count of 16.4 109 /L (4–11) with 88% neutrophils and a C-reactive level of 220 mg/L (0–5). MRI showed evidence of left hip septic arthritis with iliopsoas collection (Fig. 1). A provisional diagnosis of disseminated MSSA infection was made. Cefazolin continued, arthrotomy and washout of the joint followed by CT- guided aspiration of the iliopsoas abscess was performed on days 7 and 9 respectively from the first joint symptoms. Cloudy yellow synovial fluid containing white blood cell count 74,000/mm^3^ (86% polymorphonuclear neutrophils) was obtained from the aspirates. Subsequently, all cultures were taken back sterile. Tuberculosis and fungal infection were subsequently considered and ruled out in the aspirated samples. However, at week 3 from the first joint symptoms, fever recurred, and CRP increased to 305(0–5). Repeated MRI of the left hip showed increased joint effusion and multiple collections of the muscles around the left hip joint, mainly in the obturator internus and sartorius muscle. Hence, radiology-guided aspiration (at day 27 from the first joint symptoms) for the sartorius muscle and obturator internus was performed but persistently the cultures were negative. A second incision, drainage, and washout of the joint (at day 34 from the first joint symptoms) were performed; however, this time the MALDI-TOF isolated the organism, and specific PCR targeting the yidC gene in M. hominis confirmed the diagnosis [5]. Cefazolin was switched to Tigecycline and Moxifloxacin. Repeated MRI of the hip after three weeks revealed a significant reduction in all described abscesses coupled with improvement in clinical status in terms of fever, range of movement, and inflammatory markers (Fig. 2). Tigecycline was switched to Doxycycline, and Moxifloaxcin continued with a plan to continue therapy for 6–8 weeks. Unfortunately, the patient traveled back to his home and was lost to follow-up.

## Discussion

M. hominis was historically described by Dienes and Edsall in 1937 from Bartholin's gland abscess as the first pathogenic mycoplasma to be isolated [6]. Subsequently, M. hominis has been identified as the causative agent of several genitourinary and extragenital infections. Usually, immunocompromised hosts are at risk for extragenital infections, however, rarely cause infection in immunocompetent adults. Many risk factors have been reported for acquiring M. hominis septic arthritis such as organ transplantation, malignancies, hypogammaglobulinemia, AIDS, and immunosuppressant use including Rituximab [4], [7].

Typical symptoms, signs, and laboratory tests of septic arthritis caused by common microorganisms are present and not unique to M. hominis septic arthritis [4]. Our patient presented with typical acute native joint monoarthritis after receiving Rituximab and steroids for his recently diagnosed TTP. The joint aspiration was negative twice with evidence of progression and involvement of extra joint sites in the form of multiple muscle abscesses despite joint washout and abscess drainage. The immediate appearance of the joint symptoms after recently treated MSSA bacteremia shoved the decision toward MSSA as the possible culprit organism given the tendency to metastasize and putting in mind the cultures could be negative while patient on Cefazolin course. Notably, the IgG level was normal while a low IgM level was detected in our case. We hypothesized the genital colonization of our patient with M. hominis and disruption of the urethral mucosa by catheterization and subsequent lodging and dissemination of the organism to the hip joint and muscles.

M. hominis is fastidious and difficult to grow in ordinary cultures. Typically, agar-containing arginine media are used for isolation with the characteristic fried egg colony. Furthermore, the gram stain is usually negative due to the lack of a cell wall which further delays the identification. Therefore, various identification methods have been developed to rapidly isolate M. hominis from joint fluid [2]. Commercially available diagnostic assays for the rapid detection of M. hominis include Mycofast Revolution, Mycoplasma IST–2, and Mycoplasma Duo kit. Compared to PCR, sensitivity, and specificity of 77.3% and 80% respectively were reported for the Mycofast Revolution assay [8]. Molecular methods have revolutionized the detection of M. hominis from clinical samples to prevent the sequelae of delayed diagnosis. Conventional and Real-time PCR, 16 S rRNA, and Next-generation sequencing (NGS) have been used to diagnose M. hominis septic arthritis. However, cost constraints preclude routine use, in addition to the limitation of providing the antimicrobial susceptibility pattern of the isolates [2]. The analytical sensitivity of the molecular-based methods is 100 genome copies while culture may detect 100–1000 viable organisms per test. Of the note, the culture has 100% specificity when positive [2].

The lack of a rigid bacterial cell wall provides M. hominis with an innate resistance to all antibiotics acting on the cell wall, limiting the choice to tetracyclines, lincosamides, and fluoroquinolones. Interestingly, unlike other Mycoplasma species, M. hominis is intrinsically resistant to Erythromycin, clarithromycin, and azithromycin. However, antimicrobial susceptibility testing is not routinely performed except in the absence of clinical response to antibiotics targeting M. hominis [9]. It is worth mentioning, resistance pattern of M. hominis is varying geographically and with antimicrobial exposure in different settings throughout the world.

The optimal treatment for native joint septic arthritis due to M. hominis is not yet well defined given it is a rarity. Two active agents including fluoroquinolones, due to good penetration into the bone, have been suggested [9]. Other active agents include, Tetracyclines and Clindamycin and the duration is typically weeks to months [8]. In our case, MALDI-TOF identified the organism in only one sample but the specific M. homins PCR was positive in all. The PCR assay used in our case targeted a gene conserved in all M. hominis isolates, the yidC gene, while 16S rRNA is the target gene for conventional PCR assays [2]. The reported heterogeneity in the 16S rRNA gene of M. homins urges the search for more conservative genes like the yidC gene to further increase the sensitivity of the PCR [5]. Cefazolin was switched to Tigecycline and Moxifloxacin with an impressive clinical response in terms of fever and inflammatory markers and repeated MRI of the joint confirmed the timely radiological response.

We searched PubMed, Embase and Cochrane Library databases in June 2021 for similar cases. The search terms included “Mycoplasma,” “infection,” “joint,” and “septic arthritis,”. We excluded infection by non-M. hominis, prosthetic joint infection and children (<18 years old). The search was restricted to articles written in English and yielded a total of 15 cases of M. hominis native joint septic arthritis (Table 1). Cases ranged between 17 and 67 years of age and were predominantly female. Large joints are mostly involved, and almost half of the cases involved more than one joint. Immunocompromising conditions were seen in more than two-thirds of cases with hypogammaglobulinemia being the commonly associated risk factor. Delayed diagnoses were seen in 9 cases except in four cases where a definite diagnosis was achieved in less than 10 days. Of the 15 cases reviewed, molecular methods were used in 5 cases. Of the cases identified, only three cases reported abscesses, two of which involved the psoas muscles as in our case. Almost all cases necessitated some form of surgical procedure except in 5 cases where the only arthrocentesis was required. Doxycycline was prescribed either alone or in combination with other antibiotics in all cases except in one where Ciprofloxacin and Clindamycin were the principal therapies. The duration of therapy ranged from 4 weeks to 8 months, although the data were not always available. Two-thirds of the cases experienced complete recovery of the joint function (Table 1).

**Table 1:** Summary of previously reported adult cases of native joints septic arthritis caused by Mycoplasma hominis.

Study	Case	Gender/age, years	Affected joint	Risk factors/associated conditions	Rituximab use	Time to diagnose	Diagnostic methods	Appropriate initial antibiotics used	Type of procedure	Associated abscess	Definite antibiotic used	Duration of antibiotics	Outcome of the joints function
Verinder, 1978 [10]	1	F/40	Hip	Postpartum	No	3 days after operation	Culture	No	Joint exploration	No	xytetracycline	6 weeks	Fully recovered
McDonald et al.,1983 [11]	2	F/54	Disseminated (wrist, shoulder, knees, hip)	Non-Hodgkin’s lymphoma	No	2 months	Culture	No	Resection of the left femoral head	No	Doxycycline	NA	Partial recovered
Jorup-Rönström et al., 1989 [12]	3	F/39	Hip, Knee, shoulder	CVID/ U. urealyticum isolated also from the joint	No	13 months	Culture	No	Resection of her right femoral head	Subcutaneous abscess and ulcer	Doxycycline	4 months	Partial recovered
Clough et al., 1992 [13]	4	F/39	Disseminated (wrist, shoulder, knees)	SLE/ Low IgG	No	4 months	Culture	No	Arthroscopic debridement and drainage	No	Temafloxa and Doxycycline	8 months	Fully recovered
Luttrell et al.,1994 [14]	5	F/67	Knee	No	No	19 days	Culture	No	Arthrocentesis	Left psoas and lumbar epidural abscess	Doxycycline	35 days	Died during therapy
Franz et al., 1997 [3]	6	F/47	Knee, Wrist	Primary hypogammaglobulinemia	No	7 days	Culture	NA	NA		Doxycycline	6 months	Fully recovered
Franz et al., 1997 [3]	7	F/43	Knee, Hip, shoulder, Ankle, PIP	Primary hypogammaglobulinemia	No	NA	Culture	NA	NA but had Hip Girdlestone arthroplasty	Pyelonephritis with Psoas abscess	Ciprofloxacin + Clindamycin then Sparfloxacin	NA	Partial recovered
Garcia-Porrua et al., 1997 [15]	8	M/36	Knee	Renal transplant/HD	No	NA	Culture	NA	Open synovial biopsy with synovectomy	No	Doxycycline	NA	Fully recovered
Sendi et al., 2004 [16]	9	F/48	Knee, T12 vertebra	Primary hypogammaglobulinemia	No	22 days	PCR + Culture	No	Arthrocentesis	No	Doxycycline	NA	Fully recovered
Phuah et al., 2007 [17]	10	F/17	Hip	Postpartum	No	At least 20 days	Culture	No	Arthroscopy and wash-out	No	Doxycycline and Ciprofloxacin	12 weeks	Fully recovered
Wu et al., 2012 [18]	11	M/65	Ankle	Acute gout	No	7 days	PCR + Culture	No	Arthrocentesis	No	Doxycycline and Moxifloxacin	6 weeks	Fully recovered
Sato et al., 2012 [19]	12	M/26	Disseminated (PIP, shoulder, knees, hip)	Hypogammaglobulinemia/ Dissemination to the brain	No	At least 2 months	Culture + 16S rRNA	No	Arthrocentesis	No	NA	NA	Died
McCool 2012 [20]	13	F/27	Hip, MTP	Postpartum, CVID	No	NA	Culture	No	Arthrocentesis	No	Doxycycline	4 weeks	Fully recovered
Wynes et al., 2013 [21]	14	M/33	Ankle	X-linked agammaglobulinemia (XLA)	No	Less than 10 days	16S rRNA	No	Incision and drainage	No	Doxycycline and Moxifloxacin	8 weeks	Fully recovered
Bozo et al., 2021 [7]	15	F/58	Hip	Rheumatoid arthritis and ulcerative colitis	Yes	9 weeks	PCR + Culture	No	Incision and drainage	No	Doxycycline and Moxifloxacin	8 weeks	Fully recovered
Our case	16	M/30	Hip	TTP	Yes	5 weeks	PCR + Culture	No	Incision and drainage	Yes	Tigecycline/Doxycycline and Moxifloxacin	Planned 8 weeks	Fully recovered but lost follow up

CVID, Common variable immune deficiency; PIP, proximal interphalangeal joints; HD, Hemodialysis; MTP, metatarsophalangeal joints.

## Conclusion

Septic arthritis of the native joints caused by M. hominis is a rare clinical entity, that demonstrates the ability of the organism to manifest as an extragenital infection. In particular, immunosuppressed hosts appear at risk with the diagnostic hint provided by repeatedly negative joint aspirates. Molecular methods have revolutionized the detection of M. hominis and can prevent devastating consequences. Prompt initiation of antibiotics targeting the organism is required for a favorable outcome.

## Funding

No funding was received towards the publication.

## CRediT authorship contribution statement

**Gawahir A. Ali:** Corresponding author, clinical management, data acquisition, literature search and manuscript writing. **Wael Goravey:** Contribute to data acquisition, manuscript preparation, literature search and final proof reading. **Abdulrahman Hamad:** Clinical management. **Emad B. Ibrahim:** Contributed to data acquisition and microbiology reports. **Mohamed R. Hasan:** Contributed to data acquisition and microbiology reports. **Muna Al Maslamani:** Supervised all the aspects. **Hussam Al Soub:** Clinical management and supervised all the aspects.

## Declaration of Competing Interest

The authors declare that they have no competing interests.

## Data Availability

The authors confirm that the datasets supporting the findings of this case are available from the corresponding author upon request.
